# An Effective Method for Preparation of Liquid Phosphoric Anhydride and Its Application in Flame Retardant Epoxy Resin

**DOI:** 10.3390/ma14092205

**Published:** 2021-04-25

**Authors:** Qian Li, Yujie Li, Yifan Chen, Qiang Wu, Siqun Wang

**Affiliations:** 1 School of Engineering, Zhejiang A&F University, Hangzhou 311300, China; lyjzafu@163.com (Y.L.); yfchen.28425@foxmail.com (Y.C.); 2 Center for Renewable Carbon, University of Tennessee, Knoxville, TN 37996, USA; swang@utk.edu

**Keywords:** flame retardant, phosphorous content, liquid anhydride, epoxy resin

## Abstract

A novel liquid phosphorous-containing flame retardant anhydride (LPFA) with low viscosity was synthesized from 9,10-dihydro-9-oxa-10-phosphaphenanthrene-10-oxide (DOPO) and methyl tetrahydrophthalic anhydride (MeTHPA) and further cured with bisphenol-A epoxy resin E-51 for the preparation of the flame retardant epoxy resins. Both Fourier transform infrared spectroscopy (FT-IR), mass spectrometry (MS) and nuclear magnetic resonance (NMR) measurements revealed the successful incorporation of DOPO on the molecular chains of MeTHPA through chemical reaction. The oxygen index analysis showed that the LPFA-cured epoxy resin exhibited excellent flame retardant performance, and the corresponding limiting oxygen index (LOI) value could reach 31.2%. The UL-94V-0 rating was achieved for the flame retardant epoxy resin with the phosphorus content of 2.7%. With the addition of LPFA, the impact strength of the cured epoxy resins remained almost unchanged, but the flexural strength gradually increased. Meanwhile, all the epoxy resins showed good thermal stability. The glass transition temperature (*T*_g_) and thermal decomposition temperature (*T*_d_) of epoxy resin cured by LPFA decreased slightly compared with that of MeTHPA-cured epoxy resin. Based on such excellent flame retardancy, low viscosity at room temperature and ease of use, LPFA showed potential as an appropriate curing agent in the field of electrical insulation materials.

## 1. Introduction

Epoxy resins have been widely used in coatings, electronic materials, adhesives, and matrices for fiber-reinforced composites due to their fascinating properties such as good bonding strength, dielectric property, dimensional stability and high hardness [1,2,3,4]. However, the epoxy resins cured in the common way are flammable with a low limiting oxygen index (LOI, about 19.8%) [5,6,7], which greatly limit the application in the electric and electronic fields. Owning to such drawbacks, many efforts have been devoted to improving the flame retardant performance of epoxy resins [8,9,10]. For instance, flame retardant epoxy resins containing bromine are extensively used in the field of electronic and electrical materials because of their good flame retardancy. Nevertheless, a large amount of toxic gases was released during the combustion of these brominated epoxy resins, which would inevitably cause human poisoning and failure of escape in case of fire. Moreover, waste treatment after the working life of brominated resins may generate carcinogenic substances such as polybrominated dibenzo-p-dioxins and dibenzofurans [11,12].

Nowadays, the development of halogen-free, low toxicity, low smoke, efficient flame retardant epoxy resins has been demanded for environmental protection. Phosphorus-containing flame retardants have been widely investigated due to their good thermal stability, excellent flame retardant performance and low toxicity [13,14,15]. The metaphosphoric acid was polymerized in the combustion and the subsequent formation of a char layer retained on the surface of combustibles prevented further heat transmission. 9, 10-dihydro-9-oxa-10-phosphaphenanthrene-10-oxide (DOPO) containing biphenyl, phenanthrene ring structures and the side phosphorus group in the form of active O=P-O cyclic bond was considered to be an effective reactive flame retardant substance for epoxy resins [16,17,18]. DOPO and their derivatives exhibited good thermal stability, chemical activity and flame retardant performance because of the embedment of a phosphorus atom into the epoxy molecular chain through chemical modification [19,20,21]. Lligadas et al. [22] synthesized a phosphorus-containing fatty acid dicycloxy compound and cured with 4,4-diaminodiphenylmethane which showed good thermal stability and flame retardancy. The LOI value was 31% when the phosphorus content of the system reached 3.9%. Schäfer et al. [23] prepared two flame retardant epoxy resins with 0.81–1.66% phosphorus contents that reached UL-94V-0 level and exhibited high LOI values from 31.6 to 39.2.

Owing to the high reactivity of O=P-H bond, DOPO derivatives can be not only incorporated into epoxy matrix for the preparation of intrinsic flame retardants, but also participate as modified curing agents in the curing of epoxy resin [24,25]. Recently, DOPO-based curing agents have become a hot spot in flame retardant epoxy resins, which required lower loading to pass UL-94V-0 rating and also minimized the negative effect of additives on the mechanical properties of the cured resins. Yang et al. [26] prepared a new solid anhydride (MMDOPO) with maleinized myrcene and DOPO and then mixed MMDOPO with a maleinized organophosphorus-containing castor oil-derived diacid as curing agents for epoxy resins. The results showed the initial decomposition temperature and limiting oxygen index were elevated while the residue decreased with the increase in MMDOPO contents. Wirasaputra et al. [27] synthesized a tri-anhydride compound containing DOPO and triazine moieties and used as a halogen-free flame retardant co-curing agent for diglycidyl ether of bisphenol A/methylhexahydrophthalic anhydride system. The results showed the cured epoxy resin passed V-0 rating of UL 94 test with the limiting oxygen index of 32.7% when the phosphorus content was only 1.5 wt%. However, the DOPO-based anhydride reported in the literature generally included solid products which usually had a high melting point and were thus inconvenient to use. It is a challenge to replace the commonly liquid anhydride as a curing agent in epoxy resin casting materials for electrical insulation [28]. In this paper, a liquid phosphorous-containing flame retardant anhydride (LPFA) was synthesized from DOPO and methyltetrahydrophthalic anhydride (MeTHPA). The samples were prepared by curing bisphenol-A epoxy resin E-51 with different mass ratios between LPFA and MeTHPA. The flame retardancy, thermal response and mechanical properties of the cured epoxy resins were investigated. We believed the research will contribute meaningful information to find a facile method for the flame retardant epoxy resins.

## 2. Experimental

### 2.1. Materials

DOPO and MeTHPA (acid value about 660 mg/g, anhydride content about 43%, viscosity about 73 mPa·s (25 °C)) were purchased from Aladdin Biochemical Technology Co., Ltd., Shanghai, China. Bisphenol-A epoxy resin E-51 (YPE-618, epoxy value 0.48–0.54 mol/100 g) was supplied by Yuanbang Chemical Manufacturing Co., Ltd., Shanghai, China. *N*, *N*-dimethylbenzylamine was purchased from Sinopharm Chemical Reagent Co., Ltd., Shanghai, China.

### 2.2. Preparation of LPFA

**Scheme 1:**
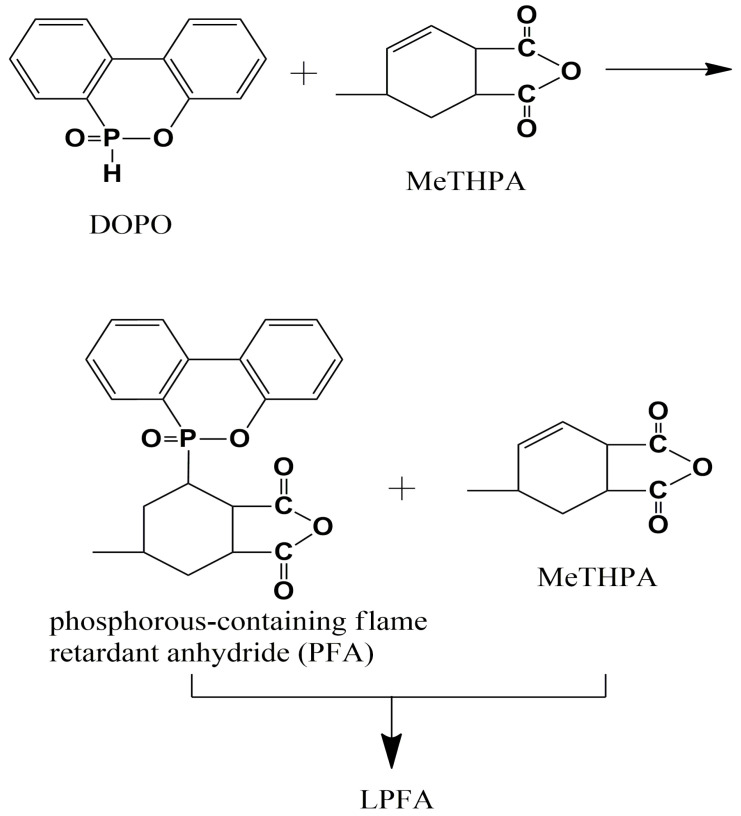
Preparation of LPFA.

An amount of 100 g (0.6 mol) of MeTHPA was added into a 500 mL four-necked flask equipped with stirrer, condenser and thermometer, and heated to 120 °C. Then, 50 g (0.2 mol) DOPO was added into the flask with constant stirring. The reactants were further heated to 180 °C and reacted for 6 h under constant stirring until a yellow transparent anhydride LPFA was obtained (Scheme 1). The MeTHPA was used in excess, so the LPFA was a mixture of phosphorous-containing flame retardant anhydride (PFA) (55.5%) and MeTHPA (44.5%). The yield of the product was about 99%. The viscosity of LPFA was 930 mPa·s (25 °C) and acid value was 506.6 mg/g. The anhydride group content was 25.72% and the phosphorus content was 4.13%.

### 2.3. Preparation of Flame Retardant Bisphenol-A Epoxy Resin

Bisphenol-A epoxy resin E-51 was cured by LPFA and MeTHPA using *N*, *N*-dimethylbenzylamine (0.5% by mass of anhydride) as an accelerator according to the stoichiometric ratio under mechanical stirring at 60 °C for 30 min. The well mixed samples were initially degassed in vacuum at 80 °C for 20 min, and then poured into the metal mold for further degassing. The samples were precured in mold at 120 °C for 2 h, demolded and post cued at 130 °C for 9 h, respectively. By altering the mass ratios of LPFA:MeTHPA over the range of 0:100, 50:50, 70:30, 86:14 and 100:0, a series of cured epoxy resins with different phosphorus contents was obtained, and the phosphorus contents of the final cured products were 0%, 1.20%, 1.74%, 2.24% and 2.70%, respectively. A control sample with the phosphorus content of 2.70% was prepared at the same conditions by adding DOPO just as a flame retardant to epoxy resin E-51\MeTHPA curing system, in which the reaction of DOPO and the epoxy network considered when calculating the stoichiometric ratio of resin and hardener.

### 2.4. Measurement and Characterizations

Acid value was measured as follows: 1 g (accurate to 1 mg) of the sample was dissolved in acetone in a 250 mL triangular flask. After adding 2–3 drops of phenolphthalein indicator, the solution was titrated with 0.1 mol/L NaOH standard solution to the end point and the acid value *A* was calculated using the Equation (1).

(1) $$A=\frac{N\times V_{1}\times 56.11}{W_{1}}$$

where: *A*—acid value, mg/g; *N*—concentration of NaOH standard solution, mol/L; *V*_1_—amount of NaOH solution consumed by sample, mL; 56.11—molecular weight of KOH; *W*_1_—mass of sample, g.

For anhydride content, a 0.3 g sample (accurate to 1 mg) was dissolved in 5 mL acetone in 250 mL iodine flask and added to 20 mL anhydrous methanol. The flask was shaken and left to stand for 5 min. Next, 3–5 drops of phenolphthalein indicator solution were added and the mixture was titrated with 0.1 mol/L NaOH standard solution.

(2) $$B=(\frac{A}{56.11}-\frac{N\times V_{2}}{W_{2}})\times \frac{72.03\times 100}{1000}$$

where: *B*—anhydride content, %; *A*—acid value, mg/g; *N*—concentration of NaOH standard solution, mol/L; *V*_2_—amount of NaOH solution consumed by sample, mL; *W*_2_—mass of sample, g; 56.11—molecular weight of KOH; 72.03—molecular weight of acetic anhydride.

The viscosity of anhydride was measured by DV-S digital viscometer (Schalod, Brookfield, MA, USA) and the temperature was 25 °C.

Fourier transform infrared (FT-IR) spectrophotometer (Bruker, Fällanden, Switzerland) was recorded in an attenuated total reflection (ATR) mode. Each sample was scanned with an average of 32 scans ranging from 4000 cm^−1^ to 500 cm^−1^.

Mass spectrometry (MS) characterization of LPFA was measured with an electrospray ionization/time of flight mass spectrometry (LCMS-IT-TOF, SHIMADZU, Kyoto, Japan) with 50% acetonitrile and 50% water as solvents under the conditions of positive ion mode spray voltage of 1.5 kV, negative ion mode spray voltage of −3.5 kV. The temperature of CDL is 200 °C. The atomized gas is nitrogen (purity ≥ 99.5%) and flow rate is 1.5 L/min. LCMS-IT-TOF was equipped with liquid chromatography, so we can separate PFA from LPFA and get the Mass spectrometry of pure PFA.

AVANCE AV-III HD 500 MHz nuclear magnetic resonance (NMR) measurement (Bruker Biospin, Fällanden, Switzerland) was carried out using deuterated chloroform as the solvent. Chemical shifts were calculated relative to tetramethyl silane (TMS) for NMR control. All ^31^P NMR spectra were tested at 298 ± 0.1 K under the conditions of a frequency of 202 MHz and scanning times of 256.

LOI values of the epoxy resins were measured on a limiting oxygen index instrument (HC-2, Jiangning Co., Nanjing, China) with a test specimen bar of 120 mm × 6.5 mm × 3.2 mm according to the standard test GB/T 2406.2-2009 [29]. Each test was repeated at least five times and the average value was reported.

Vertical combustion test of the cured products was measured with the sample size of 125 mm × 13 mm × 3.2 mm using UL94 horizontal and vertical flammability tester (KS-50B, Jinsen Co., Shanghai, China) at burning angle of 45° according to ASTM D3801. Each test was repeated at least five times and the average value was reported.

Thermogravimetric analysis (TGA) of the samples was performed with thermogravimetric analyzer (STA 409 PC/PG, Netzsch Co., Selb, Germany) under a nitrogen atmosphere from 25 °C to 800 °C at a heating rate of 10 °C min^−1^.

Differential scanning calorimeter (DSC) measurements were carried out on an instrument (Diamond, PerkinElmer, Waltham, MA, USA) from 25 °C to 180 °C at heating rate of 20 K∙min^−1^ and nitrogen gas flow rate of 20 mL min^−1^. The DSC curves were recorded the second heating cycle.

The impact strength of the cured products was tested with micro control impact testing machine (XJ-50Z, Ruike, Chengde, China) according to GB/T 2567-2008, and the dimensions of the samples were 120 mm × 15 mm × 10 mm. The flexural strength and flexural modulus were measured with CMT4304 type microcomputer controlled electronic universal testing machine (SANS Test Machine, Shanghai, China) according to GB/T 2567-2008. The test speed was set to 10 mm/min, and the dimensions of the samples were 120 mm × 15 mm × 10 mm. For each specimen, the average value was obtained from five replicates.

## 3. Results and Discussion

### 3.1. Structures Characterization with FT-IR

**Figure 1:**
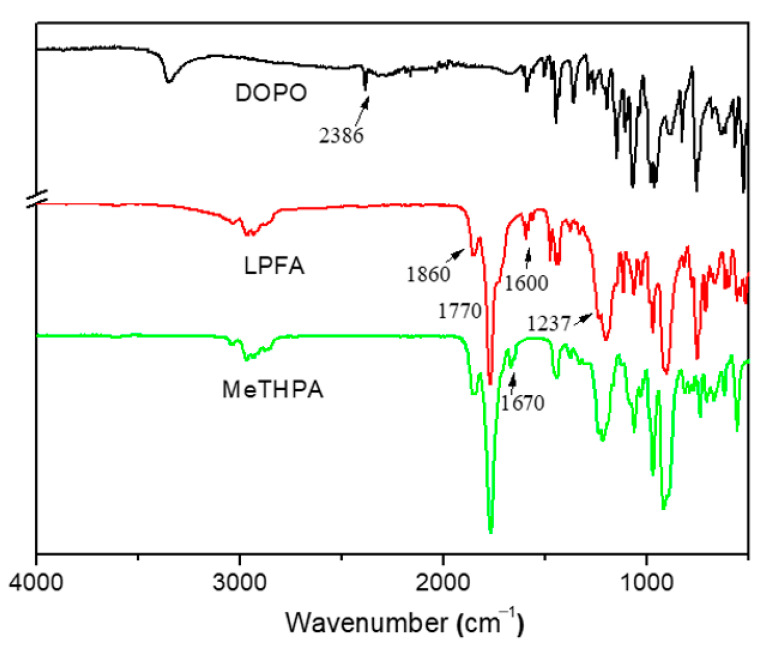
FT-IR of DOPO, MeTHPA and LPFA.

Figure 1 shows the FT-IR spectra of DOPO, MeTHPA and LPFA. In the spectrum of DOPO, an obvious peak of P-H appeared at 2386 cm^−1^ and the characteristic peak of C=C bond at 1670 cm^−1^ for MeTHPA was found. Whereas the absence of P-H peak at 2386 cm^−1^ in LPFA spectrum confirmed that LPFA had been synthesized by the reaction between the P-H bond of DOPO and the C=C bond of MeTHPA. In addition, the peaks at 1860 cm^−1^ and 1770 cm^−1^ were ascribed to -C=O stretching vibration of anhydride groups in the spectrum of LPFA. The absorption bands at 1600 cm^−1^ and 1237 cm^−1^ were attributed to benzene ring and P=O stretching vibration, which originated from the structure of DOPO, respectively [30,31].

### 3.2. MS Characterization

**Figure 2:**
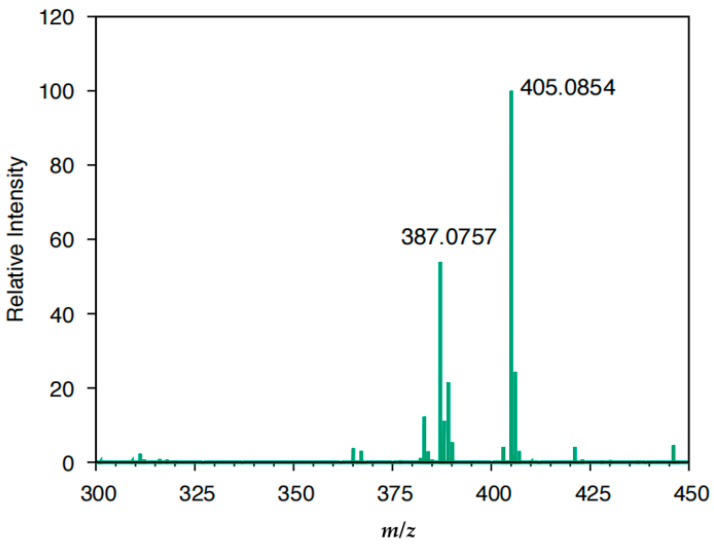
MS of PFA.

Figure 2 shows the MS of PFA (separated PFA from MeTHPA by LCMS-IT-TOF). There were two main molecular ion peaks at *m*/*z* of 387 and 405. The molecular weight of PFA is 382 and thus the peak at *m*/*z* 405 can be assigned to [M+Na]^+^. The *m*/*z* 387 was assigned to [M+Na-H_2_O]^+^, which indicated a loss of H_2_O [32]. These MS data gave a clear evidence for the formation of PFA.

### 3.3. NMR Characterization

**Figure 3:**
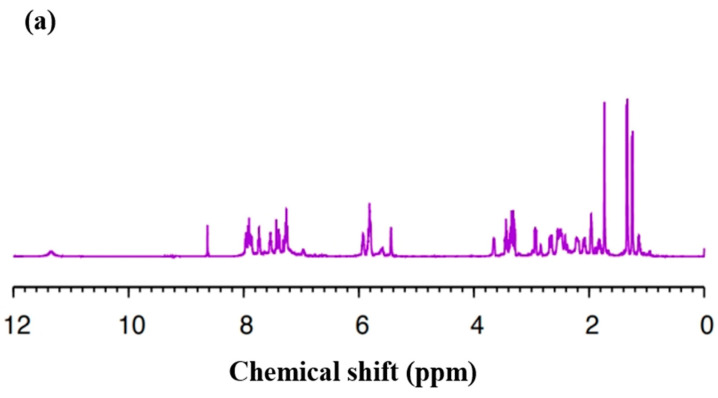

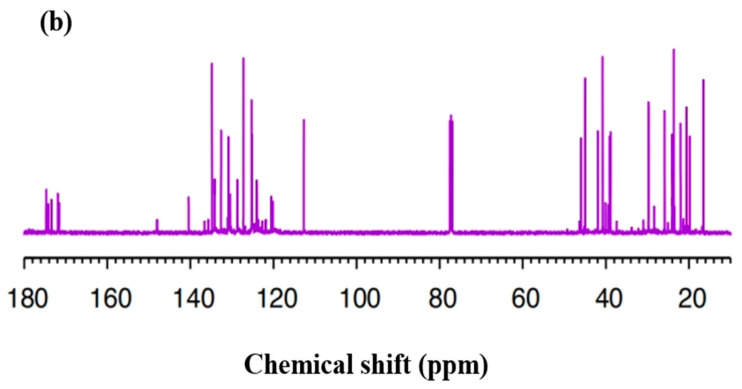
^1^H NMR (**a**) and ^13^C NMR (**b**) spectra of LPFA.

^1^H NMR profile of LPFA is shown in Figure 3a. The complete assignments of the peaks are as follows [33]: *δ* (ppm) = 6.97–7.99 for -CH=CH- in aromatic ring; 5.46–5.94 for-CH=CH- in cyclic structure; 3.24–3.45 for -CH-CO-O-; 2.53 for -CH-P-; 1.66–2.25 for -CH-C- and -CH_2_-C-; 1.11–1.32 for-CH_3_. Figure 3b shows the ^13^C NMR spectra of LPFA. The peaks at 171–174 ppm and 120–140 ppm were assigned to -CO-O- and -CH=CH- in aromatic ring, respectively. The peak of -CH-P was located at 45.7 ppm. The chemical shifts of 38.9–42.5 ppm were assigned to -CH-CO-O-, and those of 16.5–30.0 ppm were due to -CH, -CH_2_- and -CH_3_.

**Figure 4:**
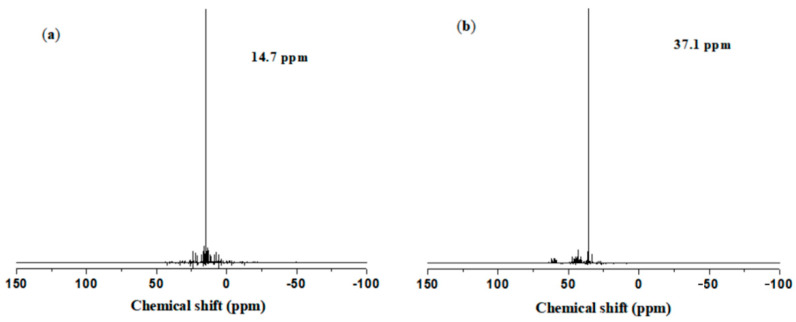
^31^P NMR spectra of DOPO (**a**) and LPFA (**b**).

^31^P NMR spectra of DOPO (a) and LPFA (b) are shown in Figure 4. The single peak of phosphorus in DOPO was at 14.7 ppm, while the peak in LPFA was at 37.1 ppm. Once DOPO was reacted with MeTHPA, P-C bond of LPFA was formed from P-H bond of DOPO. The chemical shift of P moved to low field because the electronegativity of C (2.55) was stronger than that of H (2.20). The strong electronegativity of C would reduce the density of electron cloud and the corresponding shielding effect on the shell of P atom, leading to the movement of chemical shift. These results agreed with the previous studies concerning other DOPO derivatives [34,35].

### 3.4. Flammability of Cured Epoxy Resins

Bisphenol-A epoxy resin E-51 was cured with anhydrides according to the stoichiometric ratio. The phosphorus content of cured products was changed by mixing different mass ratios of LPFA and MeTHPA. The LOI measurements and UL-94 test of the epoxy resins as a function of different phosphorus contents are listed in Table 1. The flame retardancy of epoxy resins increased gradually along with an increase in phosphorus contents. The LOI value reached 31.2 ± 0.8% for the pure LPFA-cured epoxy resin, which was much higher than that of the pure MeTHPA-cured product. The improved flame retardancy thus demonstrated that LPFA as a curing agent can impart excellent flame retardancy properties for the system. The phosphorus content required in the pure LPFA was only 2.7% to obtain such excellent flame retardant performance, whereas the bromine content must be higher than 16% to meet excellent flame retardant standards when brominated epoxy resins were used as flame retardant materials [36]. The results of UL-94 vertical combustion test are important in evaluating the upward burning characteristics. The MeTHPA-cured epoxy resin did not self-extinguish and emitted black smoke after ignition, suggesting that the normal cured epoxy resin was flammable [37]. The burning times, released smoke and flaming drip reduced as the increase in LPFA occurred. The curing agent with 50% LPFA and 50% MeTHPA resulted in a slight improvement in flame retardancy and UL-94 rating was V-2. The LPFA-cured epoxy resin reached UL-94V-0 with no dripping.

### 3.5. Thermal Stability Analysis

**Figure 5:**
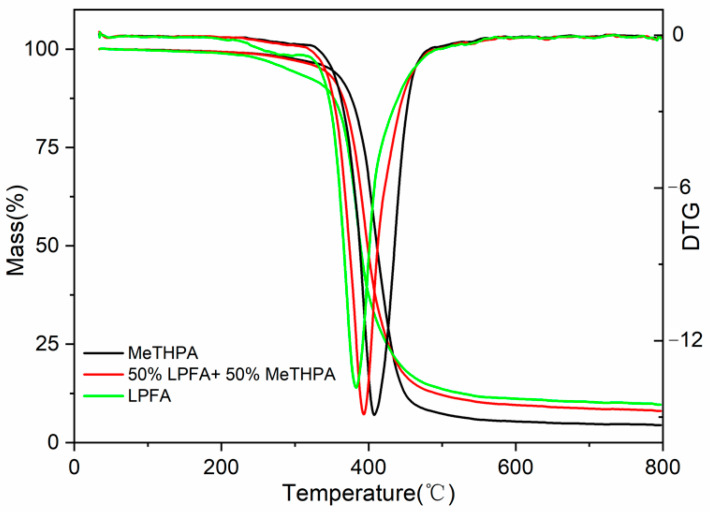
Thermogravimetric curves of the LPFA- and MeTHPA-cured epoxy resins.

Thermogravimetric (TG) curves of the LPFA and MeTHPA-cured epoxy resins are shown in Figure 5 and the related data are summarized in Table 2. All the TG curves exhibited one main plateau corresponding to the decomposition of the epoxy system. The initial decomposing temperature was decreased as the incorporation of LPFA due to its flame retardant effect by decomposition before the whole matrix decomposed. This similar phenomenon was also found for other DOPO-containing polymers [38,39]. It is important that this decrease in the initial decomposition temperature is in a particular range, which is usually shown by the flame retardant results. The peak temperature of the fastest weight loss of the LPFA-cured epoxy resin was 385 °C, while that of the epoxy resin cured with MeTHPA was 408 °C indicating that the heat resistance was slightly reduced for the phosphorus-containing epoxy resin. It can be attributed to two reasons: (1) the relatively poor thermal stability of weak P-O-C bond in the phosphaphenanthrene group, which suffered breaking in the lower temperature and subsequently formed pyrophosphate or polyphosphate substances that catalyzed the dehydration of polymeric matrix [40,41]; (2) the decrease in anhydride group contents due to the bulky structure of LPFA may cause lower crosslinking density of epoxy resins. Moreover, the char yield of the LPFA-cured resin was 11.2% at 700 °C, which was higher than the result of 4.8% obtained from the MeTHPA-cured resin. The increased residual mass of the LPFA-cured resin suggested the potential fire-retardant activity in condensed phase [31].

**Figure 6:**
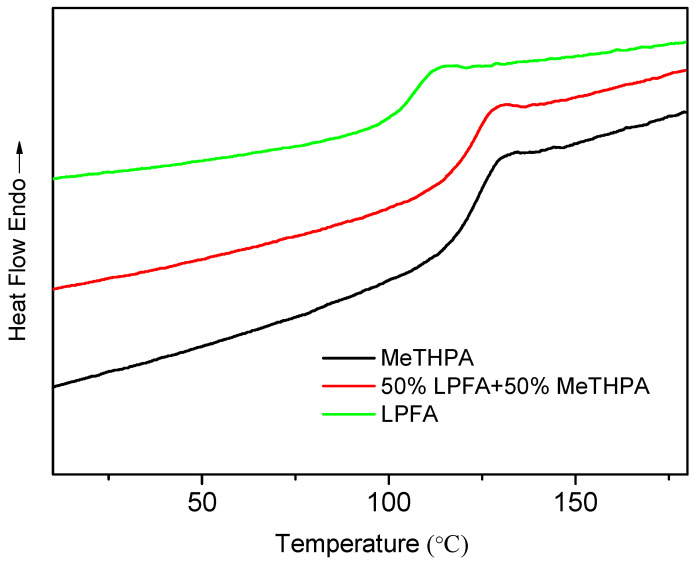
DSC of the cured epoxy resins.

Figure 6 shows the DSC curves for MeTHPA, 50% LPFA–50% MeTHPA and LPFA-cured epoxy resins. The glass transition temperature (*T*_g_) of cured epoxy resin was determined as inflection point and shown in Table 2. The *T*_g_ of the MeTHPA-cured epoxy resin was 123 °C, while that of the epoxy resin cured with LPFA was 105 °C. The *T*_g_ decreased with the addition of LPFA into epoxy resin, which can be attributed to the reduced crosslinking density of LPFA. This is also consistent with the result shown in Figure 5. *T*_g_ is an important parameter of polymer for electrical insulation. The minimum *T*_g_ of insulating materials is 90 °C according to the standard of the International Electrotechnical Commission (IEC) [42]. Therefore, *T*_g_ of the LPFA-cured epoxy resin was higher than 100 °C, which met the demand of epoxy resins for electrical insulation.

### 3.6. Mechanical Properties Analysis

**Figure 7:**
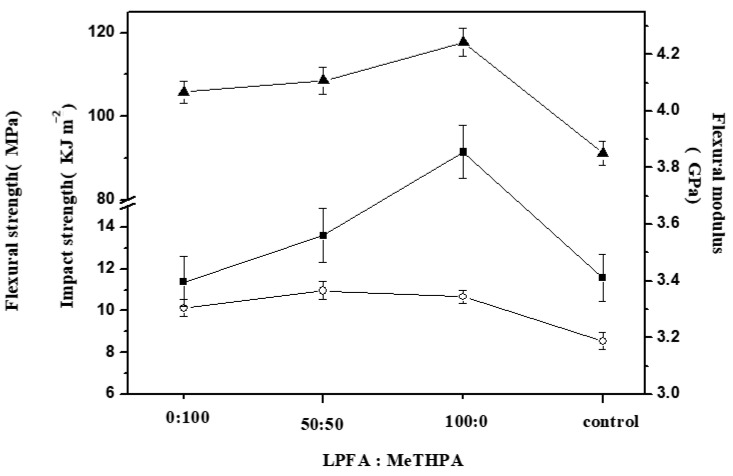
Dependence of impact strength (○), flexural strength (▲) and flexural modulus (■) of the cured epoxy resins on different anhydrides.

The impact strength, flexural strength and flexural modulus of different anhydride-cured epoxy resins are shown in Figure 7. The impact strength of the LPFA-cured epoxy resins varied from 10.12 to 10.97 KJ m^−2^, basically in the same range as that of the MeTHPA-cured resins, suggesting that the incorporation of LPFA had little effect on the mechanical properties of epoxy resins. However, the flexural strength increased from 105.8 MPa to 117.8 MPa as an increase in ratios of LPFA and MeTHPA because LPFA, as a curing agent, with bulky structure can cause longer intermolecular distance. The increase in distance between the macromolecular segments provided more space for the motion of molecular chains during curing process. The flexural modulus increased from 3.398 GPa to 3.856 GPa as an increase in ratios of LPFA and MeTHPA because LPFA contained more rigid phosphaphenanthrene ring structure compared with MeTHPA. Moreover, the control is a blank experiment with DOPO just added as a flame retardant to epoxy resin E-51\MeTHPA curing system, in which the phosphorus content was as same as the LPFA-cured epoxy resin (LPFA:MeTHPA = 100:0). The impact strength and flexural strength of the cured product decreased with the simple addition of DOPO to epoxy resin\MeTHPA curing system, suggesting that LPFA as a modified curing agent minimized the negative effect of additives on the mechanical properties in the curing of matrix resin.

## 4. Conclusions

Liquid anhydride (LPFA) with excellent flame retardancy was synthesized with DOPO and MeTHPA. Then, a series of flame retardant cured epoxy resins with different LPFA and MeTHPA contents were successfully prepared. The flame retardancy of the epoxy resins have been remarkably enhanced with the addition of LPFA. The LPFA-cured epoxy resin (LPFA:MeTHPA = 100:0) passed UL-94V-0 rating. The LOI value was 31.2% when the content of phosphorus was only 2.7%. The glass transition temperature and thermal decomposition temperature of epoxy resin cured by LPFA decreased slightly compared with that of MeTHPA-cured epoxy resin. The mechanical properties of epoxy resins cured with LPFA were increased compared with the simple addition of DOPO to the epoxy resin\MeTHPA curing system at the same phosphorus content. Liquid anhydride is convenient to use at room temperature and is more popular as a curing agent for epoxy resins. This work provided a novel method to synthesize an effective phosphorous-containing liquid anhydride for applications in flame retardant epoxy resins.

## Figures and Tables

**Table 1:** LOI measurement and UL-94 test of cured bisphenol-A epoxy resin E-51.

LPFA:MeTHPA	Phosphorus Content (%)	LOI (%)	Burning Time (s)	UL-94 Rating
0:100	0	19.9 ± 0.5	Burning all the time	-
50:50	1.20	23.9 ± 0.6	26 ± 2	V-2
70:30	1.74	26.1 ± 0.5	24 ± 2	V-2
86:14	2.24	28.3 ± 0.6	15 ± 1	V-1
100:0	2.70	31.2 ± 0.8	6 ± 1	V-0

**Table 2:** Thermal properties of the cured epoxy resins by different anhydrides.

LPFA:MeTHPA	Temperature of Decomposition Onset (°C)	Temperature of Decomposition the Fastest (°C)	*T*_g_ (°C)
0:100	355	408	105
50:50	330	392	118
100:0	312	385	123

## Data Availability

The data presented in this study are available on request from the corresponding author.
